# Periodontitis and Tooth Loss Are Associated With Higher Risks of Cognitive Disorders: A Systematic Umbrella Meta‐Analysis

**DOI:** 10.1002/cre2.70240

**Published:** 2025-11-11

**Authors:** Balen Hamid Qadir, Mohammed Khalid Mahmood, Yad Mariwan Mohammed Amin, Handren Ameer Kurda, Tara Ali Rasheed, Ahmed Hama Noori, Zana Fuad Noori, Mohammed Aso Abdulghafor, Hevi Nihad Mohammed Fadhil, Mohammed Taib Fatih, Delphine Tardivo, Herve Tassery, Romain Lan

**Affiliations:** ^1^ Department of Dentistry Komar University of Science and Technology Sulaymaniyah Kurdistan Iraq; ^2^ Faculty of Medical and Paramedical Sciences, Aix‐Marseille University, French National Center of Scientific Research (CNRS), French Blood Establishment (EFS), Bio‐Cultural Anthropology, Law, Ethics and Health Laboratory (ADES) Marseille France; ^3^ Department of Dentistry Tishk International University Sulaymaniyah Kurdistan Iraq; ^4^ College of Dentistry Sulaymaniyah University Sulaymaniyah Kurdistan Iraq; ^5^ Department of Dentistry American University of Iraq, Sulaimani (AUIS) Sulaymaniyah Kurdistan Iraq; ^6^ Department of Psychotic Disorders Sahlgrenska University Hospital Gothenburg Sweden; ^7^ Conservative and Endodontic Department Dental School of Medicine Aix‐Marseille University Marseille France; ^8^ IHU‐MEPHI Institute, Marseille Hospital APHM Marseille France

**Keywords:** Alzheimer's disease, cognitive decline, cognitive disorder, cognitive impairment, dementia, periodontal disease, periodontitis, tooth loss

## Abstract

**Objective:**

Periodontal disease (PD) and cognitive disorders (CDs) are common in older people, and these variables appear to be linked. The aim of this article was to assess this association using an umbrella meta‐analysis.

**Methods:**

PubMed and MEDLINE were searched for records until June 2025. The groups were compared using odds ratio (OR) and relative risk (RR).

**Results:**

A total of 20 meta‐analyses were included. Individuals with PD and its consequences had higher chances of having/developing all‐type CDs: periodontitis (OR = 1.65, 95% CI: 1.37–1.97, *p* = < 0.0001), tooth loss (OR = 1.51, 95% CI: 1.32–1.71, *p* = < 0.0001). The risk was much higher for patients with severe periodontitis: (OR = 2.69, 95% CI: 2.26–3.20, *p* = < 0.0001). Pooled analysis for cohort studies was (RR = 1.26, 95% CI: 1.20–1.32, *p* = < 0.0001).

**Conclusion:**

PD is associated with CDs with low to moderate certainty of evidence measured by GRADE.

## 1 Introduction

Periodontitis is the inflammation of the supporting soft and hard tissue around the dentition. Although an acute type of this inflammation can also be seen, most of the time it manifests as a chronic disease. Periodontitis is characterized by a progressive destruction of the periodontium (Savage et al. 2009). The development and progression of periodontitis are driven by a complex interplay of bacterial biofilm (plaque) and the host's immune response. Several key pathogenic microorganisms are strongly associated with periodontal disease (PD), particularly anaerobic gram‐negative bacteria. Two main groups of microorganisms are involved in the pathogenesis: *Porphyromonas gingivalis* (from the red complex group) and *Fusobacterium nucleatum* (a member of the orange complex group) (Gul et al. 2020).

PD is one of the most prevalent chronic diseases worldwide, affecting millions and contributing significantly to the global burden of oral diseases. Its distribution varies by region, age, socioeconomic status, and access to dental care. Periodontitis has a global prevalence of ~20% (Murray et al. 2020). The main risk factors of PD include poor oral hygiene, smoking, diabetes, aging, and genetic predisposition. Low‐income countries, aging populations, smokers, and diabetics have a higher risk of periodontitis (Papapanou and Susin 2017; Darby 2022).

If left untreated, periodontitis has several impacts on oral health, like pain and discomfort, tooth loss, functional impairment, and negative aesthetic and psychological effects. Periodontitis has an economic and societal burden reflected in healthcare costs, loss of productivity among patients, and reduced health‐related quality of life (Mahmood, Mohammedameen, et al. 2024; Mahmood, Fatih, et al. 2024; Al‐Sharqi et al. 2024). Despite being a local inflammation, periodontitis may cross the line of locality and can play a role in the initiation or progression of some systemic health diseases, such as cardiovascular diseases, diabetes mellitus, gastrointestinal diseases, respiratory infections like pneumonia, rheumatoid arthritis, and may even increase the risk of mortality (Mahmood, Mohammedameen, et al. 2024; Zardawi et al. 2021; Barznji et al. 2024; Seymour et al. 2007; Isola et al. 2000; Ansai et al. 2010).

Cognitive disorders (CDs), also known as neurocognitive disorders, are a category of mental health conditions characterized by significant impairments in cognitive functions like memory, learning, perception, and problem‐solving (Serrano‐Pozo et al. 2014; Wilson et al. 2020). These impairments can affect an individual's ability to function normally in daily life, and they can arise from various causes, including brain injuries, neurodegenerative diseases, frailty, and mental health conditions (Borges et al. 2019; Boyle et al. 2013). CDs primarily affect thinking, memory, and other mental processes. These disorders can make it difficult for individuals to perform everyday tasks, manage their lives, and maintain relationships. Although the exact causes of CDs are still not fully known, the risk factors are diverse: brain injuries, stroke, brain infections, some endocrine disorders, and extreme nutritional deficiencies (Petersen et al. 2014; Jakob‐Roetne and Jacobsen 2009).

The actual global data of CDs is not fully clear; however, according to the WHO, the estimated data of dementia and cognitive impairment exceeds 110 million (World Health Organization 2019). Although the classification of CDs is still debated, they are generally categorized into mild and major CDs (Petersen et al. 2014). These categories represent different levels of cognitive decline, ranging from temporary confusion to significant and progressive loss of cognitive abilities. Mild cognitive impairment (MIC) represents a noticeable decline in cognitive abilities, but the individual can still manage daily tasks independently. This may be an early stage of a more significant neurocognitive disorder (Gauthier et al. 2006). In contrast, a major CD is characterized by a significant decline in cognitive abilities that interferes with daily life and independence (Atkinson et al. 2005). It includes impairments in memory, language, executive function, and other cognitive domains. It is often progressive, meaning the cognitive decline worsens over time. Alzheimer's disease (AD) is a common example of a neurodegenerative disease that can cause dementia (Jakob‐Roetne and Jacobsen 2009; Atkinson et al. 2005).

Both PD and CDs are especially prevalent among geriatric populations (Elsig et al. 2015; Weijenberg et al. 2019). There are several research on the association between these two entities. Primary studies have investigated this association around the world among different populations. The majority of these studies point out to a positive and directly proportional association between these two variables. Recently, a number of meta‐analyses were published based on the original study data and served in the clarification of this association. Nevertheless, the findings of these meta‐analyses are heterogenous. Umbrella review is a systematic review and meta‐analysis of the meta‐analyses, and it is located at the top of the pyramid in the hierarchy of the modern evidence synthesis. As far as we searched, we could find only two umbrella reviews on the research question. With the inclusion of twelve (Lin, Chen, et al. 2024) and sixteen (Arbildo‐Vega, et al. 2025) meta‐analyses, these umbrella reviews only performed qualitative analysis without quantitative measurements. Hence the aim of this study was to provide a pooled analysis that can help clinicians and public health policy makers in their management, treatment and policy making concerning PD and CDs among geriatric population.

## 2 Materials and Methods

### 2.1 Protocol and Registration

This umbrella review was performed in accordance with the 2020 Preferred Reporting Items for Systematic Reviews and Meta‐Analyses (PRISMA) guidelines (Page et al. 2021), and relevant methodological recommendations for umbrella reviews (Fusar‐Poli and Radua 2018; Ioannidis 2009). The protocol for this review was prospectively registered in the International Prospective Register of Systematic Reviews (PROSPERO) with the registration number CRD420251065250.

### 2.2 Eligibility Criteria

The research question of this review was formulated according to PECOS/T guideline as follows: (P) Problem: Do patients with periodontitis and tooth loss have a higher risk of having/developing CDs? What is the relation between PD and different subtypes of CDs? (P) Population: Geriatric population at higher risk of PD and CDs (≥ 50 years). (E) Exposure: Periodontitis and tooth loss. (C) Comparison: Intragroup comparison between baseline and follow‐up, or intergroup comparison with the controls (or between severe and mild forms of periodontitis). (O) Outcome: CDs and their subtypes—all‐type CDs, MIC, cognitive impairment, dementia, and AD. (S) Study design: All meta‐analyses that adjusted covariates, that are published in the English language, and have presented their pooled analysis in odds ratio (OR) and relative risk (RR). (T) Time: All meta‐analyses that were published in PubMed and MEDLINE databases before June 2025.

Hence, this umbrella review included systematic reviews and meta‐analyses that examined the relationship between PD and CDs. There was no need for the definition of the variables of PD, CDs, and their subgroups, because the meta‐analyses have defined the categories when selecting their primary studies for inclusion. Hence, we just collected each category of variables together.

Since there were only observational studies on this association, the included records did not contain any experimental studies. However, there were no restrictions on the observational study design within the included reviews, whether they were cross‐sectional, case‐control, or cohort study designs.

All meta‐analyses that reported the mentioned association and presented their findings in OR and RR were included. Those few meta‐analyses, which presented their results in hazard ratio (HR) and mean difference, were excluded due to a lack of comparability. Moreover, all meta‐analyses of preclinical, animal, and in vitro studies were excluded.

Only systematic reviews and meta‐analyses published in peer‐reviewed journals in the English language were included. Reviews that did not apply systematic methods (narrative review, scoping review, etc.), those reported their results in a noncomparable statistical tool and unit, or those investigating one of the variables of PD or CDs without studying the other, were excluded. Conference abstracts, dissertations, editorials, or any other evidence not published in a peer‐reviewed journal were excluded to ensure the credibility of the evidence identified. Table 1 presents the PECOS/T formulation of the main research questions of this umbrella review.

**Table 1:** Characteristics of the included systematic reviews and meta‐analyses.

References	Number of included original studies	Study designs	Total (*N*)	Age range	Key findings	Adjusted confounders	Subgroup findings
Agarwal et al. (2024)	4	3 cohort, 1 cross‐sectional	12,079	28–79 years	Tooth loss (OR = 5.74) and periodontitis (OR = 2.99) linked to MCI. Edentulism was the strongest predictor.	Age, sex, smoking, CVD	Age > 60: OR = 3.21 (*p* < 0.01)
Asher et al. (2022)	47 (24 CD, 23 dementia)	Longitudinal cohorts	~1.3 M	≥ 50 years	PD associated with CD (OR = 1.23).	Age, sex, education, APOE4	Severe PD: HR = 1.34 (*p* = 0.02)
Chen et al. (2018)	8	5 prospective, 3 retrospective	14,362	59–107 years	Tooth loss increased dementia risk by 1.34 times; dose‐response relationship.	Age, sex, education, smoking, comorbidities	Edentulism: RR = 1.31 (*p* < 0.001)
Dibello et al. (2024)	46 (21 cognitive, 14 dementia)	Longitudinal cohorts	3,220,345	≥ 18 years	PD linked to cognitive impairment (RR = 1.25) and dementia (RR = 1.22).	Age, sex, SES, diabetes	Cognitive decline stronger in < 65 y (*p* = 0.016)
Dziedzic (2022)	17	14 cohorts, 2 case‐control, 1 cross‐sectional	311,878	> 45 years (mostly)	PD associated with dementia (OR = 1.39) and AD (OR = 1.03, *p* > 0.05). High heterogeneity (I² = 96%).	Age, sex, smoking, comorbidities	Dementia stronger in PD (*p* < 0.05); no AD link
Fang et al. (2018)	21	9 cohorts, 9 cross‐sectional, 3 case‐control	322,818	≥ 43 years	Tooth loss associated with dementia (crude OR = 2.62; adjusted OR = 1.55). Strongest in cross‐sectional studies.	Age, sex, education, vascular factors, lifestyle	Cross‐sectional: OR = 3.76 (*p* < 0.001)
Fu et al. (2024)	22	(8 cohorts, 14 cross‐sectional)	4,246,608	≥ 60 years	Periodontitis (OR = 1.45), tooth loss (OR = 1.80), and edentulism (OR = 2.07) increased the risk of cognitive impairment.	Age, sex, education, comorbidities, lifestyle	Dose‐response: 19‐28 teeth lost → OR = 2.52 (*p* = 0.005)
Guo et al. (2021)	20	8 case‐control, 4 cross‐sectional, 8 descriptive	4587	≥ 55 years	Periodontitis associated with cognitive impairment (OR = 1.77). Moderate/severe PD linked to dementia (OR = 2.13).	Age, sex, inflammation markers	CPI ≥ 3 (moderate/severe PD): OR = 2.13 (*p* < 0.05)
Hu et al. (2021)	13	5 case‐control, 5 cross‐sectional, 3 cohorts	291,114 (AD), 4805 (MCI)	≥ 54 years	PD increased AD risk (OR = 1.78) and MCI risk (OR = 1.60). Severe PD had the highest AD/MCI risk (AD: OR = 4.89; MCI: OR = 2.32).	Age, sex, comorbidities, lifestyle	Case‐control designs showed a stronger AD association (OR = 2.82)
Kaliamoorthy et al. (2022)	3	3 case‐control, 2 cohorts, 1 cross‐sectional	640	≥ 60 years	Significant association between PD and AD (OR = 1.67). Severe PD showed a stronger association (OR = 4.89).	Age, sex, smoking, comorbidities	Case‐control studies: OR = 2.70 (*p* < 0.05)
Kim and Han (2025)	24	13 case‐control, 11 cohorts	864	NP	Severe periodontitis linked to dementia (OR = 2.85) and AD (OR = 6.87). No association with moderate PD (OR = 0.94, *p* > 0.05).	Age, sex, CVD, smoking, diabetes	Severe PD + AD: OR = 6.87 (*p* = 0.04)
Larvin et al. (2023)	39	13 cross‐sectional, 26 longitudinal	1,986,194	28–100 years	PD increased cognitive decline (RR = 1.33) and dementia/AD (RR = 1.22). Severe PD showed the strongest association (RR = 1.44). Female sex amplified risks.	Age, sex, smoking, PD classification	Self‐reported PD showed lower risks (RR = 0.77–0.86)
Leira et al. (2017)	3	2 cross‐sectional, 1 cohort	~1000	≥ 60 years	Overall PD and AD: OR 1.69 (95% CI 1.21–2.35). Severe PD and AD: OR 2.98 (1.58–5.62).	Age, sex, education, smoking, comorbidities	Severe PD showed a stronger association (OR 2.98).
Li et al. (2023)	18	Prospective cohorts	356,297	≥ 45 years	Tooth loss linked to dementia (RR = 1.15) and cognitive decline (RR = 1.20). Stronger for VaD (RR = 1.25) and edentulism (RR = 1.24).	Age, sex, education, vascular factors	Asia: RR = 1.15 (*p* < 0.01); nondenture users: RR = 1.70
Lin, Pathak, et al. (2024)	7	4 case‐control, 3 cross‐sectional	3973	≥ 54 years	Periodontitis associated with MCI (OR = 1.70). Severe PD doubled MCI risk (OR = 2.09). MCI patients had worse CAL (MD = 0.44 mm).	Age, sex, education, smoking	Severe PD subgroup: OR = 2.09 (*p* < 0.001)
Nadim et al. (2020)	12	5 cohorts, 7 case‐control	225,594	20–80 years	PD associated with dementia (RR = 1.38). Cohort RR = 1.18; case‐control OR = 2.25. A 50% PD reduction could prevent 850 K dementia cases globally.	Age, sex, SES, comorbidities	Asia: RR = 1.20; Europe: RR = 1.38 (*p* > 0.05)
Qi et al. (2021)	14	9 prospective, 3 retrospective	34,074	≥ 54 years	Tooth loss increased cognitive impairment risk by 1.48 times and dementia by 1.28 times.	Age, sex, education, smoking, denture use	No denture: RR = 1.55 (*p* < 0.001)
Qiu et al. (2020)	11	4 case‐control, 5 cross‐sectional, 2 cohorts	279,963	≥ 54 years	PD associated with AD (RR = 1.22). Severe PD had a higher AD risk (RR = 1.54) versus moderate PD (RR = 1.19, *p* > 0.05). AD patients had worse PD, CAL, and plaque (all *p* < 0.05).	Age, sex, education, smoking (partial)	Severe PD subgroup: RR = 1.54 (*p* = 0.03)
Raymundo et al. (2022)	12 (5 PD, 7 tooth loss)	5 cohorts, 7 cross‐sectional	311,878	≥ 54 years	PD (OR = 3.37) and tooth loss (OR = 1.88) linked to cognitive decline.	Age, sex, smoking (partial)	Severe PD subgroup: OR = 15.12 (Holmer et al.)
Shen et al. (2016)	11	6 cohorts, 3 cross‐sectional, 2 case‐control	20,858	≥ 52 years	Tooth loss associated with increased dementia risk (RR = 1.43).	Age, sex, education, BMI	Asia: RR = 1.76 (*p* < 0.001)

Abbreviations: AD, Alzheimer's disease; BMI, body mass index; CAL, clinical attachment loss; GRADE, Grading of Recommendations Assessment, Development, and Evaluation; MCI, mild cognitive impairment; NOS: Newcastle‐Ottawa scale; NP, not reported; OR, odds ratio; PD, periodontal disease; RR, relative risk.

### 2.3 Search Strategy

To find relevant systematic reviews and meta‐analyses evaluating the relationship, the electronic databases of PubMed and MEDLINE were used to conduct the search, including all the records released before June 2025.

A search strategy was established based on a combination of controlled vocabulary (MeSH terms) and keywords related to CDs (and its subtypes), PD (and tooth loss), and review/meta‐analysis. The full list of search terms and combinations is presented in Supporting Information S1: Table 1. There were no restrictions on publication dates; however, only English articles that met the inclusion criteria were included.

### 2.4 Study Selection

All the records obtained from databases were uploaded into reference management software, and duplicates were excluded. Study selection was conducted in two parts by two independent reviewers (M. K. M. and B. H. Q.). The two‐part study selection process began (Part 1) with screening of titles and abstracts for potentially relevant studies based on eligibility criteria, and then (Part 2) the full‐text articles of potentially eligible studies were screened in detail for eligibility. Systematic reviews and meta‐analyses that met all eligibility criteria were selected for inclusion in the umbrella review. Disagreements during the screening process were resolved by discussion between the two reviewers. Where agreement was not reached, a third reviewer (M. A. A.) made the final decision.

### 2.5 Data Extraction

Data extraction was undertaken with a structured Microsoft Excel spreadsheet created for this umbrella review. Data were extracted independently by two reviewers (M. K. M. and B. H. Q.) to reduce the potential for errors and minimize variability. Discrepant data extraction was resolved through discussion, with a third reviewer (M. A. A.) consulted when consensus could not be reached.

To provide a structured collection of data by clinical variables/parameters, different worksheets within the Excel file were developed for all‐type CDs, dementia, cognitive impairment, MIC, and AD as clinical variables. The main parameters of oral health were periodontitis, tooth loss, and the severity of periodontitis.

All the CD types were collected to form a new category and called “all‐type CDs.” In addition, to see the collected effect of periodontitis and tooth loss (which is the ultimate consequence of periodontitis), another category was created and named “periodontitis + tooth loss.” This served as a dichotomy to get a general opinion on the association between PD and CDs. As there were few studies on cognitive decline, this was also collected under the category of cognitive impairment.

Study characteristics such as the number of primary studies included in the quantitative analysis, the type of study design of the primary studies, sample size, population characteristics, main statistical findings, quality of the meta‐analyses, covariate adjustments, heterogeneity, and subgroup sensitivity results were extracted.

The effect sizes for each outcome were coded as OR and 95% CI and RR with 95% CI, and they were extracted from the meta‐analytic forest plots.

### 2.6 Quality Assessment

The AMSTAR 2 (A Measurement Tool to Assess Systematic Reviews) tool was used to assess the methodological quality of the included meta‐analyses and systematic reviews (Shea et al. 2017). This is a specialized tool for evaluating the methodological quality of systematic reviews that examine studies of healthcare interventions that are either randomized or nonrandomized. The 16 items in the AMSTAR 2 tool evaluate the following domains: protocol registration, thorough literature search, evaluation of the risk of bias in individual studies, and appropriate meta‐analysis techniques. The AMSTAR 2 tool assigns a quality rating of high, moderate, poor, or critically low to each review. Special importance is given to the seven domains that AMSTAR 2 defines as “critical.” Two reviewers (M. K. M. and B. H. Q.) carried out each evaluation separately, and any disputes were settled by discussion or, if required, involvement from a third reviewer (M. A. A.).

### 2.7 Data Synthesis and Analysis

To ensure methodological consistency across included outcomes, we utilized the DerSimonian and Laird random‐effects model (DerSimonian and Laird 1986). Since all the outcomes were dichotomous, ORs and RRs with 95% CIs were recalculated.

Heterogeneity among studies was assessed using the *I*² statistical test. A *p* value of less than 0.10 was considered statistically significant for heterogeneity. The *I*² values were interpreted following the guidelines from the Cochrane Handbook for Systematic Reviews of Interventions: values between 0% and 40% were considered possibly unimportant; 30%–60% as indicative of moderate heterogeneity; 50%–90% as substantial; and 75%–100% as considerable heterogeneity (Jpt 2008).

In addition, the presence of publication bias was assessed through statistical methods. Egger's regression test was applied to detect small‐study effects, with a *p* value of less than 0.05 indicating potential bias (Egger et al. 1997). These methods provided a structured approach to assessing the robustness and reliability of the pooled estimates. All statistical analyses in this umbrella review were conducted using Cochrane's RevMan tool accessed online (Fekete and Győrffy 2025).

### 2.8 Certainty of Evidence Assessment

The GRADE (Grading of Recommendations Assessment, Development, and Evaluation) approach was used to assess the certainty of the evidence for each of the outcomes included in this umbrella review. GRADE allows for a systematic and transparent approach to assessing the quality of evidence across studies, including factors related to study design, methodological rigor, consistency of findings, directness of evidence, and imprecision (and risk of publication bias) (Guyatt et al. 2008).

Despite the data included in this review coming from meta‐analyses of observational studies, which the GRADE approach would typically categorize as “low” certainty at the beginning, we also applied the modifying factors that upgrade or downgrade the certainty in the evidence. For example, we downgraded evidence if there were outcomes with marked inconsistency in findings, serious risk of bias in the primary studies, and when effect estimates were imprecise (e.g., with large confidence intervals). Conversely, we considered upgrading the evidence if the effect was large, there was a dose–response relationship, or we felt all credible residual confounding would produce a lesser effect.

For this umbrella review, the GRADE certainty ratings reflect the certainty that there is a nonnull effect (i.e., that the true effect differs from no association) rather than certainty that there is a clinically important effect. This approach was chosen because: (1) no established minimal important differences exist for the association between PD and CDs, (2) any statistically significant association has potential public health implications given the high prevalence of both conditions, and (3) the primary goal was to establish whether an association exists rather than quantify its clinical importance.

## 3 Results

### 3.1 Study Selection

**Figure 1:**
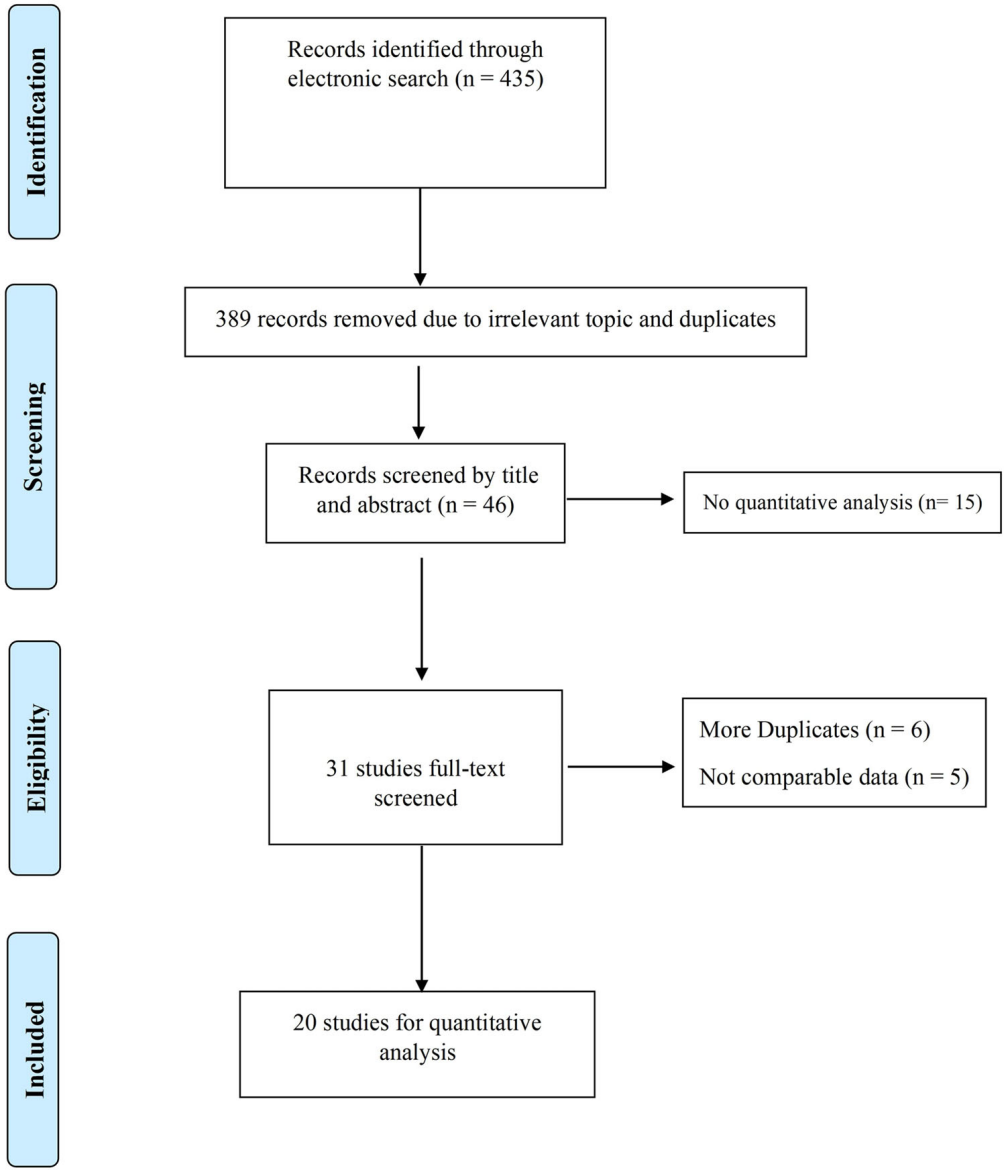
The selection process of the studies.

Through a systematic search in PubMed and MEDLINE, a total of 435 records were detected. After deleting 389 out of scope and duplicates, 46 records remained for screening. Based on our screening of titles and abstracts, we found that 15 articles did not meet the inclusion criteria due to a lack of quantitative analysis. We identified 31 articles for full‐text screening. Further, we excluded an additional six articles because the papers did not provide data relevant to the defined scope of the study (*n* = 2), were duplicates (*n* = 1), had insufficient data (*n* = 2), or were not meta‐analyses (*n* = 1). Moreover, five meta‐analyses were excluded since they presented their findings in forms other than OR and RR. Supporting Information S1: Table 2 shows these excluded studies with their reasons for exclusion. Ultimately, 20 systematic reviews and meta‐analyses met the inclusion criteria and were included in the final quantitative synthesis. The study selection process is shown in Figure 1.

### 3.2 Characteristics of the Included Systematic Review and Meta‐Analyses

This umbrella review examined the relationship between PD and CDs, mainly in the older population, including 20 systematic reviews and meta‐analyses. While study design and analytic methods varied, each systematic review followed a systematic approach to examine the literature, establishing eligibility criteria, diagnostic criteria for the respective conditions, and data synthesis for findings. The main categories of PD were periodontitis, tooth loss, and severity of periodontitis, whereas the chief categories of CDs were dementia, cognitive impairment, MIC, cognitive decline, and AD. Most of the meta‐analyses were published after 2020. In the primary studies, PD was commonly measured by clinical measures (probing pocket depth, clinical attachment loss, bleeding on probing, plaque index, gingival index, and periodontitis severity), radiographic assessment (alveolar bone loss), case definitions (CDC/AAP criteria and EFP/AAP 2018 classification), self‐reported periodontitis and tooth loss (measured by number of lost teeth or remaining teeth). On the other hand, in the primary studies, CDs were mainly measured using the following criteria: (1) clinical diagnostic criteria like DSM‐IV/DSM‐5 (Diagnostic and Statistical Manual of Mental Disorders), Criteria of National Institute of Neurological and Communicative Disorders and Stroke and the Alzheimer's Disease and Related Disorders Association (NINCDS‐ADRDA) and Internation Classification of Diseases‐10 (ICD‐10). (2) Cognitive screening tools such as the Mini‐Mental State Examination (MMSE) and Montreal Cognitive Assessment (MoCA). (3) Neuropsychological battery tests covering multiple cognitive domains (memory, attention, executive function, etc.). (4) Clinical Dementia Rating (CDR) Scale is sometimes used to rate dementia severity. (5) Self‐reported dementia.

Most of the included reviews focused on individuals ≥ 50 years. All the included meta‐analyses contained observational studies only, either cross‐sectional, case‐control, or longitudinal cohorts. The range of primary study inclusion among the meta‐analyses was broad, with a minimum of 3 and a maximum of 47 primary studies. In total, 411 primary studies were included in all the meta‐analyses. However, 70 (20%) of these were overlapping and duplicated records. The meta‐analyses covered approximately 10 million participants. Again, due to duplicate studies, this sample size may decrease to approximately 7–8 million. Concerning the longitudinal studies, the follow‐up duration ranged from 5 to 32 years. Age, sex, education, comorbidities, lifestyle, and socioeconomic status were the most adjusted covariates among the included meta‐analyses. Table 1 presents the key characteristics of these studies.

### 3.3 Overlapping Studies

Since the majority of the included meta‐analyses were published recently and in a short time span, it was expected to have a high overlapping ratio in terms of the included unique and primary studies. Indeed, it turned out that 1 in every 5 included primary studies overlapped between the meta‐analyses. Supporting Information S1: Table 3 presents the number and ratio of the overlapping studies.

### 3.4 Quality of Systematic Reviews and Meta‐Analyses

The AMSTAR 2 instrument was utilized to evaluate the methodological quality of the systematic reviews and meta‐analyses included. Out of the 20 included meta‐analyses, 7 scored “moderate” quality, 6 had “low” quality, 4 received “critically low” quality, while only 3 studies scored a “high” quality assessment.

None of the meta‐analyses reported on the sources of funding of their included studies (Q10). Only 20% of the meta‐analyses provided a list of excluded studies and justified the exclusions (Q7). Half of the studies registered their protocol beforehand in databases (Q2). Many studies did not or partially considered the risk of bias in individual studies when interpreting/discussing the results of the review (Q13). Again, many of the review authors did not or partially carried out an adequate investigation of publication bias (Q15). Supporting Information S1: Table 4 provides a full itemized AMSTAR 2 rating for each included meta‐analysis.

### 3.5 Association Between PD and All‐Type CDs

We performed a total of 12 pooled analyses. Detailed results are presented in Table 2.

**Table 2:** Umbrella meta‐analysis results.

Exposure	No. of included estimates	Effect size (95% CI)	*p* value	*I* ^2^, *p* heterogeneity	p Egger's test (Publication bias)	GRADE
*All‐type cognitive disorders (OR)*						
Periodontitis	16	1.65 (1.37, 1.97)	< 0.0001	91%, < 0.0001	Nearly 0	Low
Tooth loss	6	1.51 (1.32, 1.71)	< 0.0001	72.7%, 0.0026	0.60	Moderate
Periodontitis + tooth loss	22	1.60 (1.41, 1.83)	< 0.0001	91%, < 0.0001	Nearly 0	Low
Severe periodontitis	8	2.69 (2.26, 3.20)	< 0.0001	14.5%, 0.31	0.30	High
*All‐type cognitive disorders (RR)*						
Periodontitis + tooth loss	13	1.26 (1.20, 1.32)	< 0.0001	65.4%, 0.0005	0.004	Low
*Dementia (OR)*						
Periodontitis + tooth loss	5	1.56 (1.34, 1.81)	< 0.0001	49%, 0.097	0.77	Moderate
*Dementia (RR)*						
Periodontitis + tooth loss	7	1.26 (1.18, 1.34)	< 0.0001	63.7%, 0.01	0.024	Low
*Cognitive impairment (OR)*						
Periodontitis + tooth loss	8	1.54 (1.32, 1.80)	< 0.0001	74.6%, 0.0003	0.024	Low
*Cognitive impairment (RR)*						
Periodontitis + tooth loss	5	1.31 (1.19, 1.45)	< 0.0001	70%, 0.009	0.043	Low
*Alzheimer's disease (OR)*						
Periodontitis + tooth loss	5	1.52 (1.06, 2.18)	0.02	84%, < 0.0001	0.01	Very low
*Alzheimer's disease (RR)*						
Periodontitis + tooth loss	3	1.20 (1.13, 1.26)	< 0.0001	23%, 0.27	0.38	Moderate
*Mild cognitive impairment (OR)*						
Periodontitis + tooth loss	4	1.98 (1.43, 2.74)	< 0.0001	85%, 0.0001	0.42	Low

*Note:* GRADE ratings reflect certainty that the true effect is a nonnull effect rather than certainty of clinical importance.

Abbreviations: CI, confidence interval; GRADE, Grading of Recommendations Assessment, Development, and Evaluation; OR, odds ratio; RR, risk ratio.

**Figure 2:**
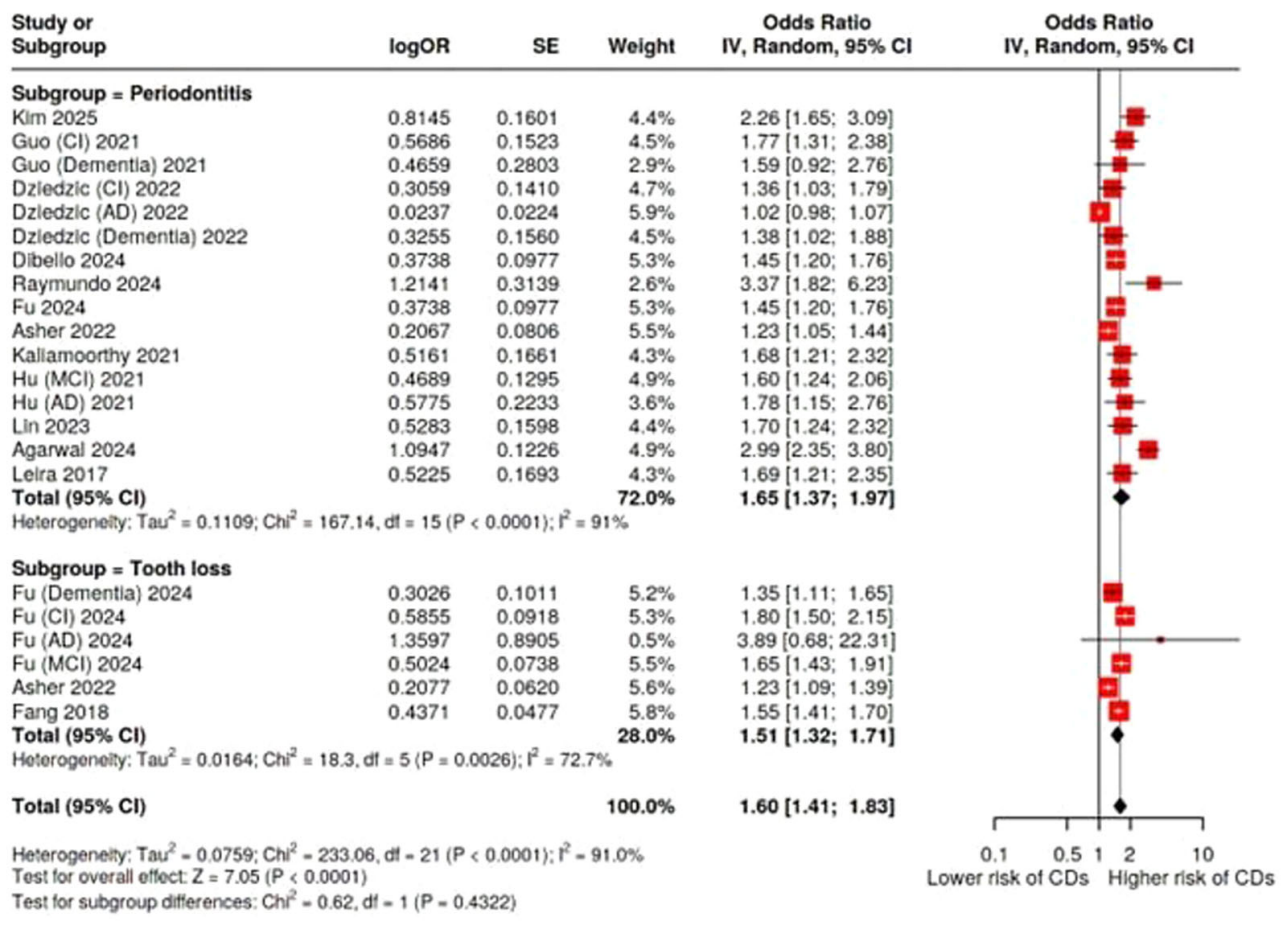
Forest plot of the association of periodontitis and tooth loss with all‐type cognitive disorders. AD, Alzheimer's disease; CI, cognitive impairment; MCI, mild cognitive impairment.

**Figure 3:**
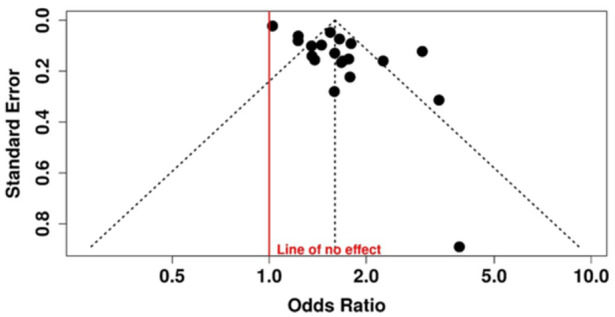
Funnel plot of the association between collected periodontitis and tooth loss with all‐type cognitive disorders showing significant publication bias (measurement: OR).

Sixteen effect sizes from 12 meta‐analyses were included that examined the relationship between PD and all‐type CDs. The pooled analysis demonstrated a statistically significant association, and people with periodontitis were more likely to have all‐type CDs than people without periodontitis (OR = 1.64, 95% CI: 1.36–1.98, *p* < 0.0001). There was high and statistically significant heterogeneity across the meta‐analyses (*I*² = 91%, *p* < 0.0001). Moreover, evidence of publication bias was present (*p* = nearly 0). Based on the GRADE framework, the certainty of the evidence for this association was rated as low. Concerning tooth loss, pooled analysis of six estimates from three studies was significantly different: (OR = 1.51, 95% CI: 1.32–1.71, *p* < 0.0001). The heterogeneity was substantial and significant (*I*² = 73%, *p* = 0.002), but no publication was observed (*p* = 0.60). The quality of the evidence of this outcome was rated as “moderate” according to GRADE. When all the studies on periodontitis and tooth loss were collected and compared with all‐type CDs, the pooled OR from 22 estimates and 13 studies was 1.60 (95% CI: 1.40–1.83), and this result was statistically significant (< 0.0001). However, both heterogeneity (*I*² = 91%, *p* = 0.001) and publication bias were significant. GRADE score for this outcome was “low” (Table 2, Figures 2 and 3).

**Figure 4:**
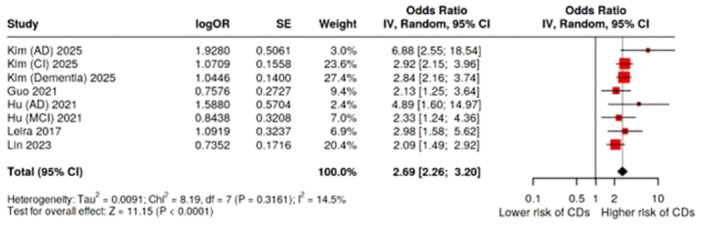
Forest plot of the association between severe periodontitis and all‐type cognitive disorders. AD, Alzheimer's disease; CI, cognitive impairment.

Five studies and eight estimates compared severe with mild to moderate periodontitis in all‐type CDs. Severe periodontitis was significantly associated with all‐type CDs (OR = 2.69, 95% CI: 2.26–3.20, *p* < 0.0001). The heterogeneity among the studies was possibly unimportant (*I*² = 14.5%, *p* = 0.31) and Egger's test showed no proof of publication bias (*p* = 0.30). Therefore, the GRADE quality for this outcome was rated as “high” (Table 2, Figure 4).

Eight included meta‐analyses and 13 effect sizes presented their findings in RR. The results of this pooled meta‐analysis revealed a “low” quality of evidence measured by GRADE. Even though the effect size was very significant (RR = 1.22, 95% CI: 1.16–1.28, < 0.0001). However, the heterogeneity was substantial (*I*² = 62.7%, 0.006) and the publication bias was nearly significant (*p* = 0.055) (Table 2, Supporting Information S1: Figures 5 and 6).

### 3.6 Association Between PD and CD Subtypes

#### 3.6.1 Dementia

Nine meta‐analyses examined the levels of periodontitis and tooth loss among people diagnosed with and without dementia. The pooled analysis showed higher odds of dementia among people with periodontitis and tooth loss. Both of the effect sizes of OR and RR were higher than 1 and were statistically significant, but evidence of heterogeneity and publication bias was observed. Hence, pooled analysis for OR and RR received “moderate and “low” quality of evidence, respectively, when assessed by GRADE (Table 2, Supporting Information S1: Figure 7).

#### 3.6.2 Cognitive Impairment

Twelve studies assessed the cognitive impairment outcome in patients with periodontitis compared with healthy periodontium. Eight estimates from five meta‐analyses measured their outcome in OR, compared with four meta‐analyses and five effect sizes for RR. Despite the statistical significance of the pooled analyses, both findings were rated as “low” using the GRADE tool (Table 2 and Supporting Information S1: Figure 8).

#### 3.6.3 MIC

Four meta‐analyses investigated the outcome of MIC in periodontitis cases and healthy periodontium controls. The certainty of this pooled analysis received a “moderate” rank measured by GRADE: (OR 1.98, 95% CI: 1.43, 2.74, *p* < 0.0001; Heterogeneity: 85%, *p* = 0.0001; Publication bias: Egger's test: *p* = 0.42) (Table 2 and Supporting Information S1: Figure 9).

#### 3.6.4 AD

Eight meta‐analyses compared periodontitis and tooth loss among individuals with AD. Pooled OR in five meta‐analyses was: (OR = 1.52, 95% CI: 1.06, 2.18, *p* = 0.02; Heterogeneity: 84%, *p* = < 0.0001; Publication bias: *p* = 0.043). The overall certainty of evidence for this outcome was rated as “very low.” Conversely, the pooled analysis of RR studies was significant and gained a “moderate” rate of evidence quality according to GRADE tool: (RR = 1.20, 95% CI: 1.13, 1.26, *p* < 0.0001; Heterogeneity: 23%, *p* = 0.27; Publication bias: *p* = 0.38) (Table 2 and Supporting Information S1: Figure 10).

All the OR results of this review should be interpreted cautiously, as ORs may overestimate effects when outcomes are common (> 10%), as is the case with CDs in geriatric populations.

## 4 Discussion

The results of this comprehensive umbrella meta‐analysis offer strong support for the notion that there is a significant correlation between CDs and PD. The findings demonstrate that those with PD had higher chances of experiencing cognitive deterioration, with strong correlations especially seen in cases of severe periodontitis. Low heterogeneity and publication bias in important subgroups (such as severe periodontitis) and the stability of these findings across various study designs and demographics reinforce the validity of the associations.

Despite the statistical significance of all 12 pooled analyses of this review, the relations of PD with all‐type CDs and their categories were mostly rated “low” to “moderate” using the GRADE framework. This was due to the vast heterogeneity among the studies and high levels of publication bias. However, we found a stronger result (OR = 2.69, GRADE: High) for severe periodontitis, indicating a dose–response relationship, that is, people with severe periodontitis have higher odds of CDs when compared with mild/moderate periodontitis. Nevertheless, it is worth noting that only five studies and eight effect sizes contributed to this result. Normally, different levels of clinical PD are expected to have different impacts. However, not investigating this dose–response relation in most of the included meta‐analyses is certainly a drawback.

Among the included meta‐analyses, three of them reported dose–response analysis. According to Chen et al.'s dose–response meta‐analysis, each lost tooth may raise the risk of dementia by 1.01 times (RR = 1.01, 95% CI = 1.00–1.02) in a linear model. Additional subgroup analyses revealed that individuals with tooth loss who do not wear dentures may be more susceptible to dementia than those who do (RR = 0.98, 95% CI = 0.87–1.10 for denture wearers; RR = 1.53, 95% CI = 1.19–1.97 for nondenture wearers) (Chen et al. 2018). Compared with older persons with high dentition, those with reduced dentition were more likely to experience cognitive impairment. When comparing the number of teeth lost (19–28 vs. 0–18 teeth) (OR = 2.52, 95% CI: 1.32–4.80, *p* = 0.005) and (28 vs. 0–27 teeth) (OR = 2.07, 95% CI: 1.54–2.77, *p* < 0.001), subgroup analysis showed that older people with fewer remaining teeth were more likely to develop cognitive impairment than those with more remaining teeth (Fu et al. 2024). A 0.011 higher relative risk of dementia and a 0.014 higher relative risk of cognitive impairment were linked to each extra tooth loss. Participants who were edentulous had a 1.40‐fold increased risk of receiving a dementia diagnosis and a 1.54‐fold increased risk of cognitive impairment (Qi et al. 2021). The literature reported the same dose–response relationship pattern. For instance, the high residual teeth number group was linked to a roughly 50% lower incidence of dementia than the low residual teeth number group (pooled OR = 0.483). However, because of the considerable heterogeneity among the included studies, the overall quality of evidence was graded as extremely low (Oh et al. 2018). In another study, the odds were 1.27 times higher for those who lost teeth without dentures than for those who had dentures. The authors came to the conclusion that prompt and cost‐effective denture restoration could help avoid cognitive decline (Ma et al. 2025).

Normally, the effect size of RR is more reliable than OR due to several reasons, such as (1) interpretability and clinical relevance: RR is more intuitive and directly interpretable as it compares the probability of the outcome of CDs between two groups (exposed to periodontitis vs. unexposed). In contrast, OR, especially when outcomes are common (> 10%), can overestimate the effect size compared with RR, making it less reliable for clinical interpretation. This overestimation can introduce uncertainty, lowering the GRADE rating. (2) Study design and outcome frequency: RR is typically derived from cohort studies, which are prospective and less prone to certain biases. These studies often provide higher‐quality evidence due to their longitudinal nature. However, OR is commonly used in case‐control studies, which are retrospective and more susceptible to biases (e.g., selection bias, confounding). (3) Clinical and mechanistic plausibility: RR studies may better capture temporal causality (periodontitis → chronic inflammation → CDs pathology over years); but OR studies might include reverse causation (early CDs → poor oral hygiene → periodontitis), weakening the association.

Indeed, we observed a clear pattern of this overestimation in all the pooled analyses. Pooled OR and RR for all‐type CDs were 1.60 and 1.26, respectively. For dementia, polled OR and RR were 1.56 and 1.26, respectively. The results of cognitive impairment analysis were 1.54 and 1.31 for OR and RR, respectively. Concerning AD, pooled OR and RR were 1.52 and 1.20, respectively. This clearly shows that cohort studies are more reliable in the investigation of this association. Importantly, we discovered that there was no expected pattern for RR to always receive higher GRADE ratings than OR analyses. For example, all‐type CDs for OR and RR were rated “low,” while dementia received “moderate” with OR compared with “low” with RR. This variability from the expected pattern probably represents the complex interaction of different levels of quality of study designs, heterogeneity, and the possibility of publication bias within the different subsections of primary studies informing our OR versus RR, and thus supporting the rationale of needing to look at multiple measures of effect in a meaningful way when interpreting the conclusions from umbrella reviews.

The methodological quality of the included meta‐analyses, as evaluated by the AMSTAR 2 tool, varied significantly; 7 scored “moderate” quality, 6 had “low” quality, 4 received “critically low”, while only 3 studies scored a “high” quality assessment. High‐quality reviews Dibello et al. (2024), Kim and Han (2025), Qi et al. (2021) demonstrated rigorous adherence to AMSTAR 2 criteria, including protocol registration (PROSPERO), comprehensive literature searches (≥ 5 databases + gray literature), and robust risk‐of‐bias assessments (e.g., ROBINS‐I, NOS). These studies also addressed heterogeneity through subgroup analyses and meta‐regression, enhancing the reliability of their findings. In contrast, critically low‐rated reviews lacked protocol registration, had incomplete risk‐of‐bias adjustments, or failed to assess publication bias quantitatively. Notably, only 3 out of 20 included meta‐analyses fully conducted sensitivity analyses to evaluate the impact of bias on results, a critical limitation given the observational nature of the primary studies. The inherent biases and reported deficiencies in the lower‐quality meta‐analyses might have led to some imprecision in our pooled estimates, potentially affecting the generalizability of the review's conclusions. These methodological limitations collectively suggest that our pooled estimates should be interpreted with caution, and the true associations between PD and CDs may be weaker than our findings suggest. The variability in quality underscores the need for stricter adherence to systematic review guidelines, particularly in protocol registration, bias mitigation, and transparency in reporting, to strengthen future evidence syntheses in this field.

We tried to conduct a sensitivity analysis based on the quality of the included studies. However, we found mixed results; in some cases, the high and moderate quality studies gave a lower estimate, while in some other cases, the critically low and low‐quality studies revealed higher pooled estimates. We believe this comes from the vast heterogeneity among the included studies in terms of the definition of the variables, measurement modes, differences in follow‐up times, sample size, and not adjusting for shared risk factors and covariates.

The literature supports an association between PD and CDs across different study designs. For example, a systematic review of preclinical studies showed that in situ brain manifestations and systemic effects are caused by oral or systemic exposure to periodontopathogens or their byproducts. The brain and serum had significantly higher levels of cytokines, amyloid peptides (Aβ), and their derivatives. Furthermore, tau protein hyperphosphorylation, hippocampus microgliosis, and neuronal death were noted in infected mice. Memory deterioration resulted from exposure to periodontal infection, which adversely affected cognitive behavior (Salhi et al. 2023). Regarding the relationship between oral microbiota and CDs, Liu et al. discovered that the presence of oral bacteria (OR = 10.68) and *P. gingivalis* (OR = 6.84) in the brain was associated with an elevated risk of AD by more than 10 and 6 times, respectively. Although the alpha diversity of oral microbiota was lower in AD patients than in healthy controls, the results of several investigations on bacterial communities varied. According to the best evidence synthesis, there is a moderate amount of evidence supporting both the general link between oral bacteria and AD and the idea that oral bacteria increase the risk of AD (Liu et al. 2023).

A Mendelian randomization (MR) analysis revealed no direct causal link between AD and PD. On the other hand, the probability of AD development was considerably elevated by tooth loss. The negative causal link between AD and denture restoration provided additional support for these findings. The authors came to the conclusion that occlusion function could be protective in preserving brain health and that its impairment could eventually lead to AD (Wang et al. 2024). Another MR study that examined the relationship between AD and many diseases found that AD and chronic periodontitis had genetic connections through five single‐nucleotide polymorphisms (OR = 1.10; 95% CI: 1.02–1.19; *p* = 0.013). Cardiovascular characteristics, such as hypertension (OR = 4.30; *p* = 0.044) and coronary artery disease (OR = 1.07; *p* = 0.021), were, however, causally associated with an increased risk of AD (Zhu et al. 2025).

It is believed that a number of interrelated biological and behavioral mechanisms contribute to the association between periodontitis/tooth loss and CDs. The main directions that are supported by current research are listed as follows: (1) systemic inflammation and neuroinflammation: local chronic inflammation caused by periodontitis may activate pro‐inflammatory cytokines like TNF‐α, CRP, and IL‐6, eventually leading to systemic inflammation (Wang et al. 2019; Said‐Sadier et al. 2023). After reaching the circulation, cytokines may pass the blood–brain barrier. In addition, chronic inflammation may trigger the immune system in the brain, consequently damaging the neurons (Gil Montoya et al. 2020). Moreover, amyloid plaque, which is a defining feature of AD, can be increased by inflammatory mediators (Nascimento et al. 2019; Teixeira et al. 2017). Indeed, the presence of *P. gingivalis* DNA and the harmful enzymes called gingipains in postmortem human AD brains has been established (Dominy et al. 2019; Siddiqui et al. 2019; Kanagasingam et al. 2020; Olsen and Singhrao 2019). (2) Direct reach of pathogens and bacteria to the brain: During eating or dental operations, oral bacteria, such as *P. gingivalis* and *F. nucleatum*, can enter the bloodstream and cause bacteremia. In addition, these pathogens may pass via peripheral nerves such as the trigeminal nerve and enter the brain (Al‐Obaidi and Desa 2018; Pisani et al. 2022). Gingipains from *P. gingivalis* degrade neuronal proteins, leading to the formation of Tau tangles and amyloid‐β plaques (Nara et al. 2021; Olsen and Singhrao 2020). Besides, the complement system can be activated by bacterial toxins, exacerbating neurodegeneration (Eslami et al. 2023). *P. gingivalis*‐specific antibodies have been found in postmortem AD brains (Dominy et al. 2019; Poole et al. 2013) and have been demonstrated to lessen amyloid pathology in mice (Zhang et al. 2025). (3) Vascular damage and hypoperfusion: Periodontitis is associated with endothelial dysfunction and atherosclerosis. Reduced cerebral blood flow from vascular injury causes mixed pathology and vascular dementia. Long‐term hypoxia resulting from vascular injury speeds up neuronal death (Rubio et al. 2020; Bezerra et al. 2024). White matter hyperintensities, which are MRI indicators of small artery disease, are linked to tooth loss in some studies (Carballo et al. 2023; Del Brutto et al. 2016; Aarabi et al. 2019). (4) Nutritional deficits and chewing dysfunction: tooth loss affects mastication, which results in poor diet and undernutrition (Jung et al. 2022). Furthermore, chewing activates parts of the brain linked to memory (Ono et al. 2010). On the other hand, edentulism has been linked to a smaller hippocampus (Kobayashi et al. 2018). (5) Shared covariates: Diabetes, hypertension, and smoking are common comorbidities that raise the risk of PD and CDs (Lee et al. 2020; Marruganti et al. 2023). The composition of the oral microbiome may also be influenced by the APOE4 allele, which is a gene also associated with an increased risk of AD (Singhrao et al. 2017). (6) Disturbance in the gut‐brain axis: oral dysbiosis increases systemic inflammation and modifies the gut microbiota (Sansores‐España et al. 2021). Neuroinflammation may be exacerbated by this “leaky gut” because it allows bacterial metabolites, such as lipopolysaccharides, to enter the bloodstream (Adil et al. 2025; Adnan et al. 2025). In summary, the complex association between periodontitis and CDs includes chronic inflammation, direct bacterial invasion, vascular damage, nutritional deficits, shared risk factors, and a disturbed gut‐brain axis.

Through integrating the propelling evidence from the literature and the findings of this umbrella review, which can be defined as demographically global as it contains data from approximately 7 million people around the world, we wanted to test whether this association is strong enough to be called causality or not. For this reason, we used Bradford Hill's Criteria (Hill 1965). This tool utilizes a multilevel scoring system for causation, which mainly includes the strength of the association, temporality, consistency, biological plausibility, specificity, and experimental evidence. Although the evidence partially fulfills several criteria (strength, consistency, plausibility), the lack of experimental and interventional data and certain temporality prevent a definitive causation (Bach 2019). Detailed assessment of each domain is presented in Table 3.

**Table 3:** Causal relationship assessment using Bradford‐Hill's Criteria for the association between periodontal disease and CDs.

	Bradford‐Hill's Criteria
1. Strength of Association:	Support: Moderate associations (e.g., OR = 1.65 for all‐type CDs; OR = 2.69 for severe periodontitis).
	Limitation: Associations are statistically significant but modest in magnitude.
3. Consistency:	Support: Multiple studies across different populations report similar associations.
	Limitation: High heterogeneity and publication bias values indicate variability, but the direction of the effect is consistent.
5. Specificity:	Limitation: Periodontitis is linked to multiple systemic conditions, so the association is not specific to cognitive decline.
	Also, specificity is less critical for multifactorial diseases like CDs.
7. Temporality:	Support: Cohort studies in the meta‐analysis suggest periodontitis precedes cognitive decline.
	Limitation: Few studies track periodontal status and cognitive outcomes over long periods. Moreover, approximately half of the studies were not longitudinal.
9. Biological Gradient (Dose‐Response):	Support: Severe periodontitis shows stronger associations (OR = 2.69) than mild cases.
	Limitation: Not all studies were stratified by periodontitis severity.
11. Plausibility:	Support: Proposed mechanisms (like systemic inflammation and bacterial toxins crossing the blood–brain barrier) are biologically plausible.
	Limitation: Studies show *P. gingivalis* can exacerbate CD pathology in mice, but human studies are scarce.
13. Coherence:	Support: Aligns with known links between chronic inflammation and neurodegeneration.
	Limitation: No direct evidence that treating periodontitis prevents CDs.
15. Experiment:	Limitation: No randomized trials show that periodontal treatment reduces the risk of CDs.
16. Analogy:	Support: Similar inflammatory pathways are implicated in other chronic diseases (e.g., diabetes, cardiovascular disease).
Conclusion	The evidence partially fulfills several criteria (strength, consistency, temporality, plausibility), but the lack of experimental/interventional data precludes definitive causation.

Abbreviation: CD, cognitive disorder.

To translate the findings of this review into the language of public health measures, we performed a population attributable risk (PAR) analysis to estimate the number of CD cases attributable to PD. For this purpose, we chose the pooled analysis of PD in all‐type CDs (RR: 1.26 (1.20–1.32)). According to the Global Burden of Disease 2019, approximately 1.1 billion (11%) people experienced severe periodontitis, and South Asia had the highest prevalence rates, with 17.5% (Murray et al. 2020). Hence, we took 20% for the global prevalence of all‐type periodontitis. For the global data of CDs, according to WHO, the estimated data of dementia and cognitive impairment exceeds 110 million (World Health Organization 2019). Therefore, we took 100 million as a reference for the global number of CD cases. The PAR results showed that with the current estimation of 20% prevalence of periodontitis, approximately 5% (5 million cases) can be attributed to PD. If the prevalence of periodontitis is reduced by half to become 10%, this can save 2.5 million people from CDs. If the prevalence is reduced to 5%, another 1 million people could be protected from CDs. In their meta‐analysis, Nadim et al. reported a PAR of 3.47% corresponding to 1.7 million cases of dementia attributable to PD (Nadim et al. 2020). Table 4 shows the estimated calculation of the PAR analysis.

**Table 4:** Estimated number of cognitive disorder cases attributable to periodontal disease in the world.

Prevalence of periodontal disease	Relative risk	Population attributable risk (%)	Number of attributable cognitive disorder cases in millions
20% (Current estimation)	1.26 (1.20–1.32)	4.94 (3.85%–6.01%)	5 (4–6)
If reduced to 10%	1.26 (1.20–1.32)	2.53 (1.94%–3.18%)	2.5 (2–3)
If reduced to 5%	1.26 (1.20–1.32)	1.32 (1.01%–1.63%)	1.3 (1–1.6)

Although the included meta‐analyses of this review contained definitions of PD and CDs when choosing primary studies for inclusion, we recognize that there was inconsistency in those definitions across the synthesized evidence. In particular, definitions of PD ranged from clinical measures of PD (such as probing depth, clinical attachment loss, and bleeding on probing) to self‐reported PD or dental records. Definitions of CD ranged from clinical diagnoses that followed evidence‐based criteria or practices (such as DSM‐5 or ICD‐10 classifications) to standardized cognitive assessment tools (MMSE or MoCA) or neuropsychological batteries. Variation in definitions for diagnostic criteria creates a level of heterogeneity in our pooled analyses, and we suggest that more future research into PD and CDs make use of standardized diagnostic definitions to help comparisons between findings be more accurate and generalizable.

This study had some limitations. First, reverse causality—cognitive decline resulting in oral hygiene—remains a credible alternative hypothesis, as the observational character of the included research prevents causal inference. Second, residual confounding cannot be ruled out even after controlling for common confounders (such as age, smoking, and comorbidities). Third, substantial heterogeneity was observed in the definitions of PD and cognitive outcomes in several studies.

Especially from a methodological point of view, for future systematic reviews and meta‐analyses, we recommend the following points: comprehensive literature searches including at least three databases plus gray literature, standardized risk‐of‐bias assessment using appropriate tools, quantitative assessment of publication bias using multiple methods (Egger's test, funnel plots, trim‐and‐fill), detailed reporting of funding sources for all included primary studies, provision of complete lists of excluded studies with justification, and subgroup analyses by PD severity and CD subtypes. Moreover, for future primary studies, we suggest the following recommendations: adoption of standardized PD definitions (e.g., 2017 World Workshop classification), use of validated cognitive assessment tools with established cut‐points, prospective cohort designs with minimum 5‐year follow‐up periods, comprehensive confounder adjustment including inflammatory biomarkers, investigation of dose–response relationships, assessment of effect modification by age, sex, and APOE genotype.

In conclusion, this comprehensive meta‐analysis summarizes strong evidence that connects PD to CDs, with the degree of periodontitis showing up as a crucial variable. Although longitudinal studies indicate a moderate but steady and consistent correlation, it is still not fully clear what mechanisms underlie this link, whether they are caused by shared risk factors, microbial translocation, or systemic inflammation. In the absence of more interventional research, our results support the inclusion of oral health therapies in geriatric care as a possible means of reducing cognitive decline. To further elucidate the causal relationship and guide therapeutic treatment, future research should give priority to prospective designs, standardized exposure assessments, and biological mechanism investigations.

## Author Contributions

**Balen Hamid Qadir:** data curation, investigation, validation. **Mohammed Khalid Mahmood:** conceptualization, investigation, methodology, visualization, writing – original draft, writing – review and editing. **Yad Mariwan Mohammed Amin:** data curation, investigation, validation. **Handren Ameer Kurda:** investigation, methodology, validation. **Tara Ali Rasheed:** conceptualization, supervision, methodology, writing – original draft, writing – review, and editing. **Ahmed Hama Noori:** methodology, investigation, software, writing – original draft, Writing – review and editing. **Zana Fuad Noori:** data curation, investigation, validation. **Mohammed Aso Abdulghafor:** resources, writing – original draft, writing – review and editing. **Hevi Nihad Mohammed Fadhil:** investigation, methodology, validation. **Mohammed Taib Fatih:** resources, writing – original draft, writing – review and editing. **Delphine Tardivo:** conceptualization, methodology, supervision, project administration. **Herve Tassery:** conceptualization, methodology, supervision, project administration. **Romain Lan:** conceptualization, methodology, supervision, project administration.

## Ethics Statement

The authors have nothing to report.

## Consent

The authors have nothing to report.

## Conflicts of Interest

The authors declare no conflicts of interest.

## Supporting information

Supporting information.

## Data Availability

Data supporting this study are available in the Supporting Information.
