# Supplementary material for: Periodontitis and Tooth Loss Are Associated With Higher Risks of Cognitive Disorders: A Systematic Umbrella Meta‐Analysis
Source: Clin Exp Dent Res. 2025 Nov 11;11(6):e70240. doi: 10.1002/cre2.70240 (PMC12606017; doi:10.1002/cre2.70240)
Supplement: Supplementary file 1 — Supporting information. [file CRE2-11-e70240-s001.docx]

**Supplementary material for the article:**

**Periodontitis and tooth loss are associated with higher risks of cognitive disorders: A systematic umbrella meta-analysis**

| **Supplementary Table 1. The search strategy of the umbrella meta-analysis** | |
| --- | --- |
| **Periodontal disease** | “periodontal disease” OR “periodontitis” OR “periodontal health” OR “tooth loss” OR “tooth loss count” OR “tooth number” OR “tooth count” OR “remaining tooth count” |
| **Cognitive disorders** | "cognitive disorder" OR “cognitive impairment” OR “cognitive impair” OR “cognitive decline” OR “dementia” OR “Alzheimer’s disease” OR “Alzheimer” OR “Alzheimer’s” |
| **Meta-analysis** | “meta-analysis” OR “Meta-analysis” OR “meta analysis” OR “Meta analysis” OR "Meta-Analysis" OR "meta-analyses" OR "Meta-Analysis" OR "meta-analyze" OR "Systematic Review" OR "Systematic Reviews as Topic" OR "Meta-Analysis as Topic" |
| **Combination** | (“periodontal disease” OR “periodontitis” OR “periodontal health” OR “tooth loss” OR “tooth loss count” OR “tooth number” OR “tooth count” OR “remaining tooth count”) AND ("cognitive disorder" OR “cognitive impairment” OR “cognitive impair” OR “cognitive decline” OR “dementia” OR “Alzheimer’s disease” OR “Alzheimer” OR “Alzheimer’s”) AND (“meta-analysis” OR “Meta-analysis” OR “meta analysis” OR “Meta analysis” OR "Meta-Analysis" OR "meta-analyses" OR "Meta-Analysis" OR "meta-analyze" OR "Systematic Review" OR "Systematic Reviews as Topic" OR "Meta-Analysis as Topic") |

| **Supplementary Table 2. Excluded meta-analyses**   \| **Study** \| **Reason of exclusion** \| \| --- \| --- \| \| Gusman et al. 2018 [30] \| Outcome measured in mean difference \| \| Maldonado et al. 2018 [31] \| Outcome measured in mean difference \| \| Dioguardi et al. 2019 [32] \| Outcome measured in hazard ratio \| \| Cerruti-Kopplin et al. 2016 [33] \| Outcome measured in hazard ratio \| \| Oh et al. 2018 [34] \| Compared low and high residual teeth number groups \|   **Supplementary Table 3. Ratio of overlapping included primary studies among the meta-analyses**   \| **Meta-analyses** \| **No. of included primary studies** \| **No. of overlapping primary studies** \| \| --- \| --- \| --- \| \| Agarwal (2024) \| 4 \| 4 \| \| Asher (2022) \| 47 \| 7 \| \| Chen (2018) \| 8 \| 1 \| \| Dziedzic (2022) \| 7 \| 2 \| \| Dibello (2024) \| 45 \| 15 \| \| Fang (2018) \| 21 \| 3 \| \| Fu (2024) \| 22 \| 4 \| \| Guo (2021) \| 20 \| 4 \| \| Hu (2021) \| 13 \| 2 \| \| Kaliamoorthy (2021) \| 3 \| 1 \| \| Kim (2025) \| 24 \| 3 \| \| Larvin (2023) \| 39 \| 4 \| \| Leira (2017) \| 5 \| 1 \| \| Li (2023) \| 21 \| 3 \| \| Lin (2023) \| 7 \| 2 \| \| Nadim (2020) \| 12 \| 3 \| \| Qi (2021) \| 14 \| 3 \| \| Qiu (2020) \| 11 \| 2 \| \| Raymundo (2024) \| 7 \| 3 \| \| Shen (2016) \| 11 \| 3 \| \| Total \| **341** \| **70 (overlapping ratio: 20.5%)** \|   **Supplementary Table 4. Methodological quality of included systematic reviews and meta-analyses using AMSTAR 2** | | | | | | | | | | | | | | | | | |
| --- | --- | --- | --- | --- | --- | --- | --- | --- | --- | --- | --- | --- | --- | --- | --- | --- | --- | --- | --- | --- | --- | --- | --- | --- | --- | --- | --- | --- | --- | --- | --- | --- | --- | --- | --- | --- | --- | --- | --- | --- | --- | --- | --- | --- | --- | --- | --- | --- | --- | --- | --- | --- | --- | --- | --- | --- | --- | --- | --- | --- | --- | --- | --- | --- | --- | --- | --- | --- | --- | --- | --- | --- | --- | --- | --- | --- | --- | --- | --- | --- | --- | --- | --- | --- | --- | --- | --- | --- | --- | --- | --- | --- | --- | --- | --- |
| **Author [ref]** | **Q1** | **Q2 (C)** | **Q3** | **Q4**  **(C)** | **Q5** | **Q6** | **Q7 (C)** | **Q8** | **Q9 (C)** | **Q10** | **Q11 (C)** | **Q12** | **Q13 (C)** | **Q14** | **Q15 (C)** | **Q16** | **Level of evidence** |
| Agrawal [1] | Y | Y | N | Y | Y | N | Y | Y | Y | N | PY | N | PY | Y | N | Y | Low |
| Asher [2] | Y | N | Y | Y | Y | N | N | Y | Y | N | Y | PY | Y | Y | N | Y | Critically low |
| Chen [3] | Y | N | Y | Y | Y | Y | PY | Y | N | N | Y | N | N | Y | PY | Y | Critically low |
| Dibello [4] | Y | Y | Y | Y | Y | Y | PY | Y | Y | N | Y | PY | Y | Y | Y | Y | High |
| Dziedzic [5] | Y | N | Y | Y | N | N | PY | Y | Y | N | Y | PY | Y | Y | Y | Y | Moderate |
| Fang [6] | Y | N | Y | Y | Y | Y | PY | Y | Y | N | Y | PY | PY | Y | Y | Y | Moderate |
| Fu [7] | Y | N | Y | Y | Y | Y | N | Y | Y | N | Y | PY | PY | Y | Y | Y | Low |
| Guo [8] | Y | N | Y | Y | Y | Y | PY | Y | Y | N | Y | PY | Y | Y | PY | Y | Moderate |
| Hu [9] | Y | N | Y | Y | Y | Y | PY | Y | Y | N | Y | PY | PY | Y | N | Y | Low |
| Kaliamoorthy [10] | Y | Y | Y | Y | Y | Y | PY | Y | Y | N | Y | PY | PY | Y | Y | N | Moderate |
| Kim & Han [11] | Y | Y | Y | Y | Y | Y | Y | Y | Y | N | Y | Y | Y | Y | Y | Y | High |
| Larvin [12] | Y | Y | Y | Y | PY | N | N | Y | Y | N | Y | Y | Y | Y | Y | Y | Moderate |
| Leira [13] | Y | Y | Y | Y | Y | Y | PY | Y | Y | N | Y | PY | PY | Y | N | Y | Low |
| Li [14] | Y | Y | Y | Y | Y | Y | N | PY | Y | N | Y | PY | PY | Y | PY | PY | Low |
| Lin [15] | Y | Y | Y | Y | Y | Y | PY | Y | Y | N | Y | PY | PY | Y | N | Y | Low |
| Nadim [16] | Y | N | Y | Y | Y | Y | N | Y | Y | N | Y | PY | Y | Y | Y | Y | Moderate |
| Qi [17] | Y | Y | Y | Y | Y | Y | PY | Y | Y | N | Y | Y | Y | Y | Y | Y | High |
| Qiu [18] | Y | N | Y | Y | Y | Y | N | Y | PY | N | Y | N | N | PY | N | Y | Critically low |
| Raymundo [19] | Y | Y | Y | Y | Y | PY | N | Y | Y | N | Y | PY | PY | Y | N | Y | Moderate |
| Shen [20] | Y | N | Y | Y | N | N | N | Y | N | N | Y | N | N | Y | PY | Y | Critically low |
| **(C): critical domain, ref: references, PY: partially yes, N: No, Y: yes. Q1: Did the research questions and inclusion criteria for the review include the components of PICO?, Q2: 2. Did the report of the review contain an explicit statement that the review methods were established prior to the conduct of the review and did the report justify any significant deviations from the protocol?; Q3, Did the review authors explain their selection of the study designs for inclusion in the review?; Q4, Did the review authors use a comprehensive literature search strategy?; Q5, Did the review authors perform study selection in duplicate?; Q6, Did the review authors perform data extraction in duplicate?; Q7, Did the review authors provide a list of excluded studies and justify the exclusions?; Q8, Did the review authors describe the included studies in adequate detail?; Q9, Did the review authors use a satisfactory technique for assessing the risk of bias?; Q10, Did the review authors report on the sources of funding?; Q11, Did the review authors use appropriate methods for statistical combination of results?; Q12, Did the review authors assess the potential impact of RoB in individual studies on the results?; Q13, Did the review authors** **account for RoB in individual studies when interpreting/ discussing the results of the review?; Q14, Did the review authors provide a satisfactory explanation for, and discussion of, any heterogeneity?; Q15, Did the review authors carry out an adequate investigation of publication bias?; Q16, Did the review authors report any potential sources of conflict of interest?** | | | | | | | | | | | | | | | | | |


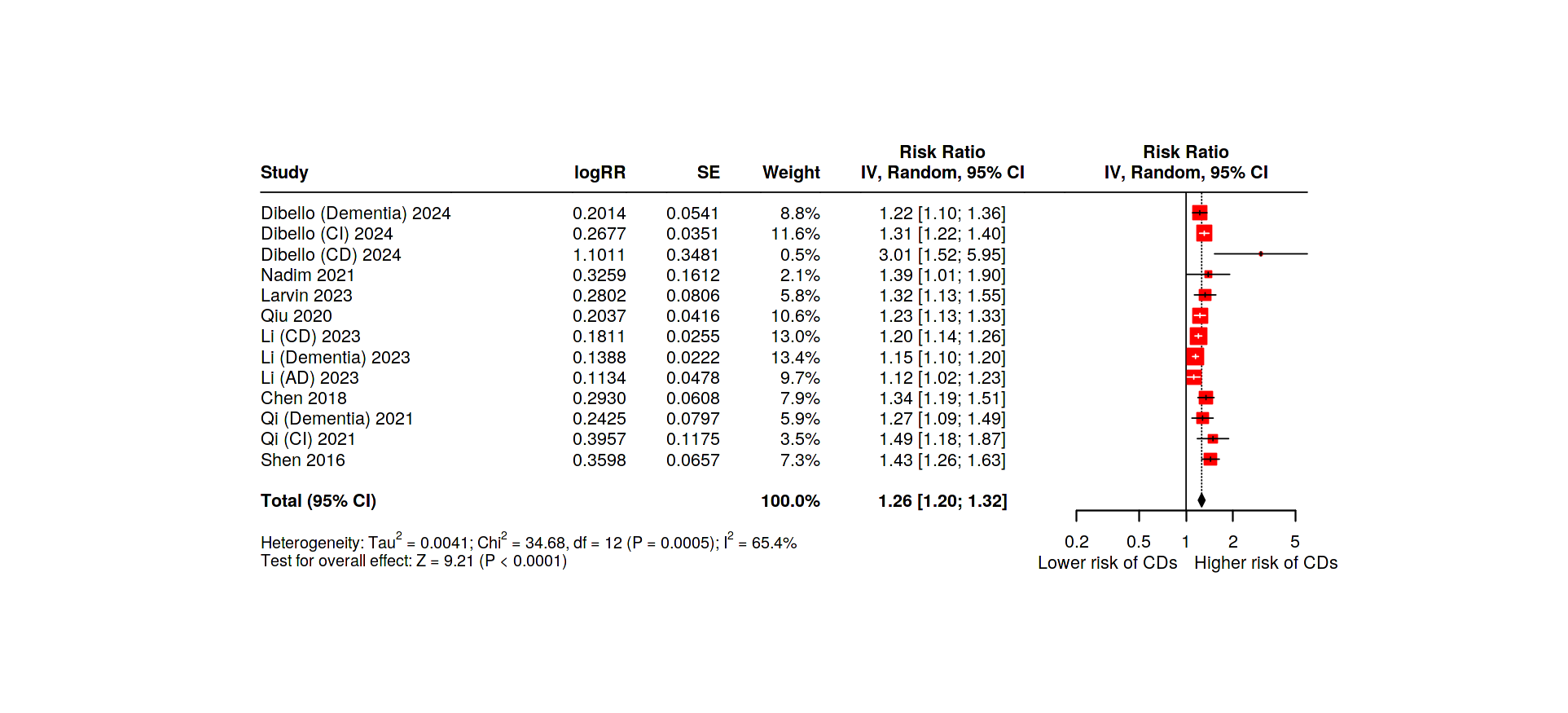


**Supplementary Figure 5.** Forest plot of the association between collected periodontitis + tooth loss with all-type cognitive disorders measured in RR.

AD: Alzheimer’s disease, CI: cognitive impairment, CD: cognitive decline


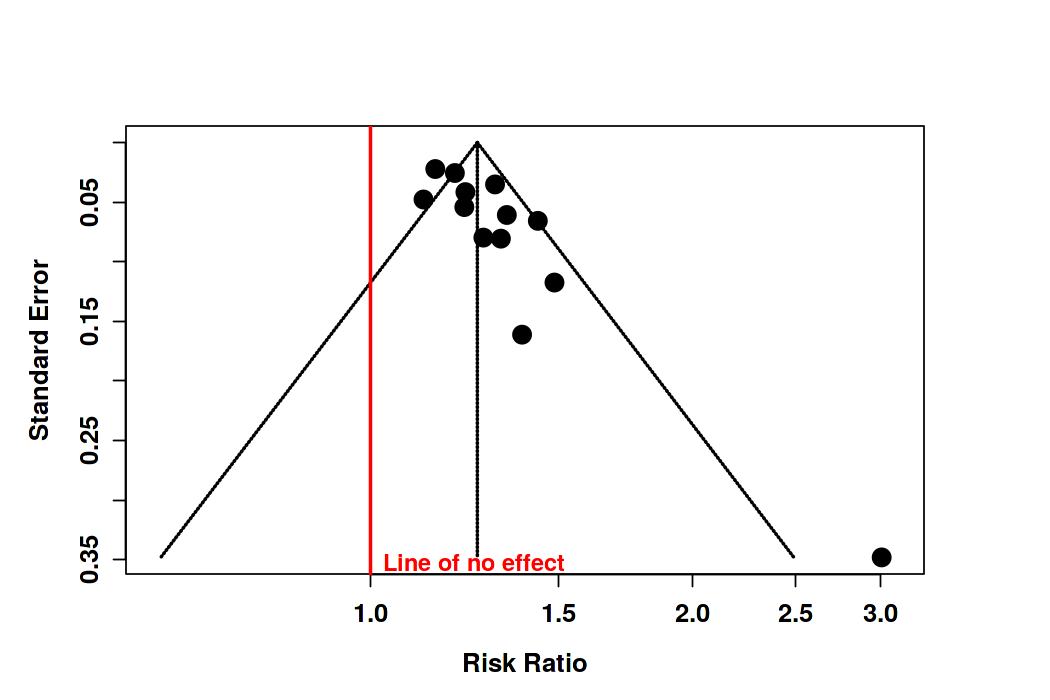


**Supplementary Figure 6.** Funnel plot of the association between collected periodontitis and tooth loss with all-type cognitive disorders showing significant publication bias (measurement: RR).


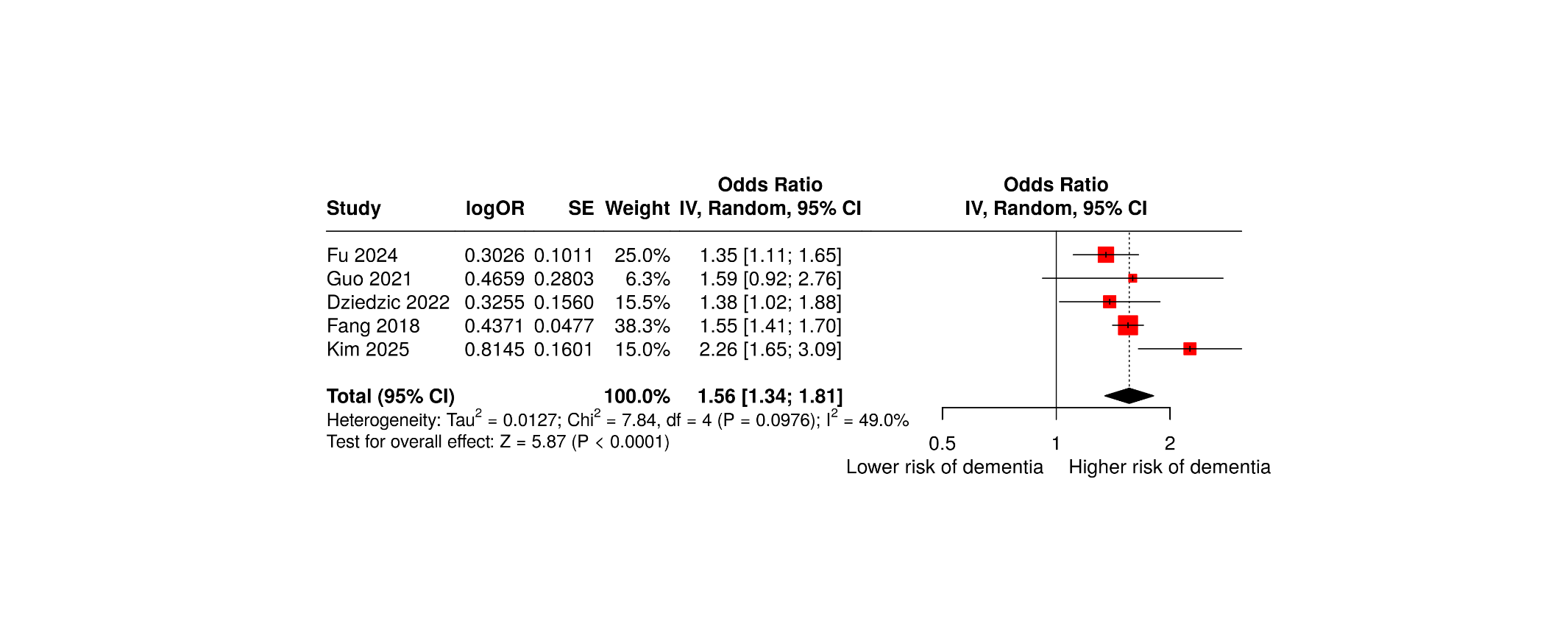


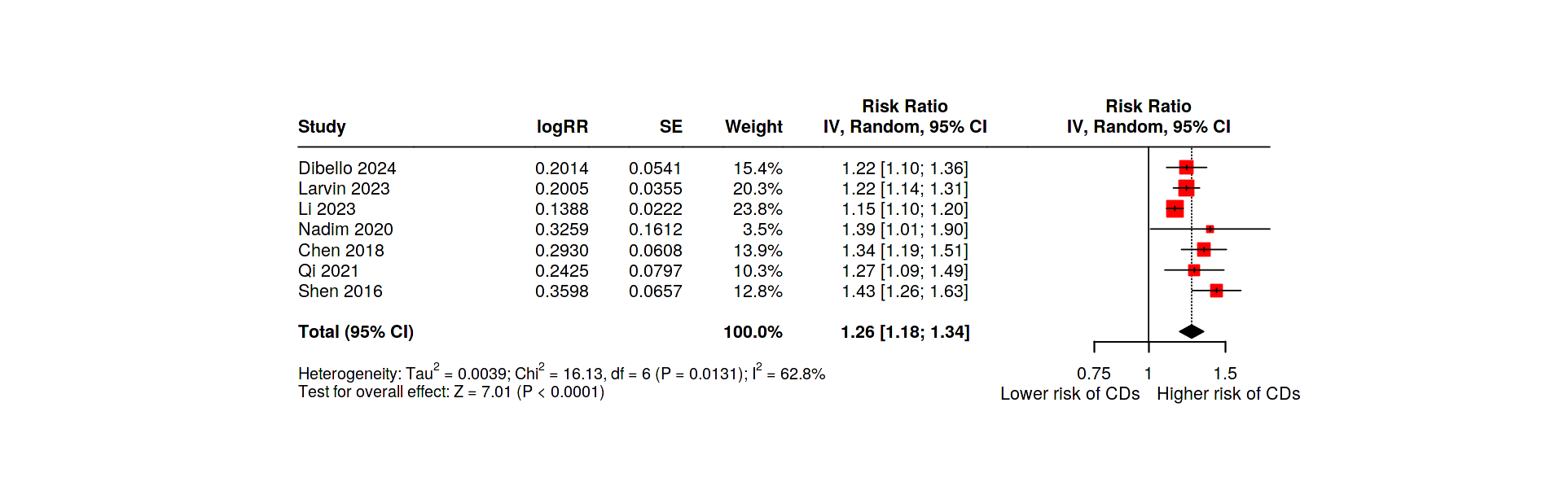


**Supplementary Figure 7.** Forest plot of the association between collected periodontitis + tooth loss and dementia measured in OR and in RR.


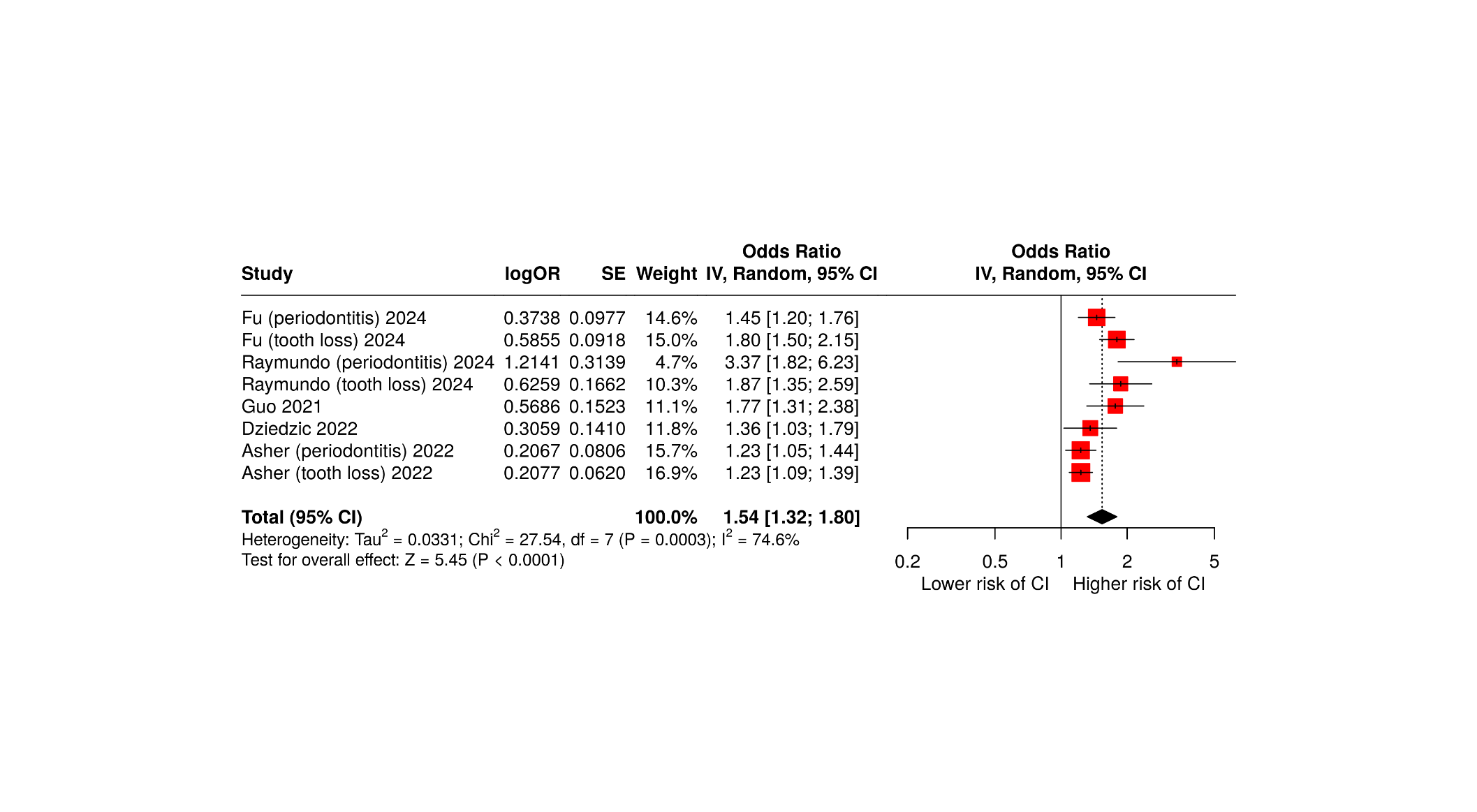


**
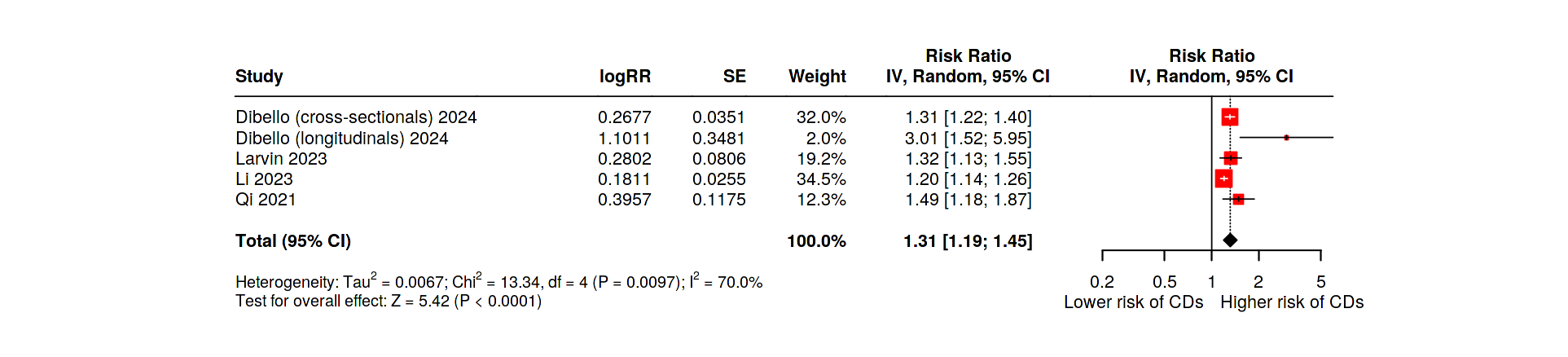
**

**Supplementary Figure 8.** Forest plot of the association between periodontitis + tooth loss and cognitive impairment in OR and RR.

CI: cognitive impairment


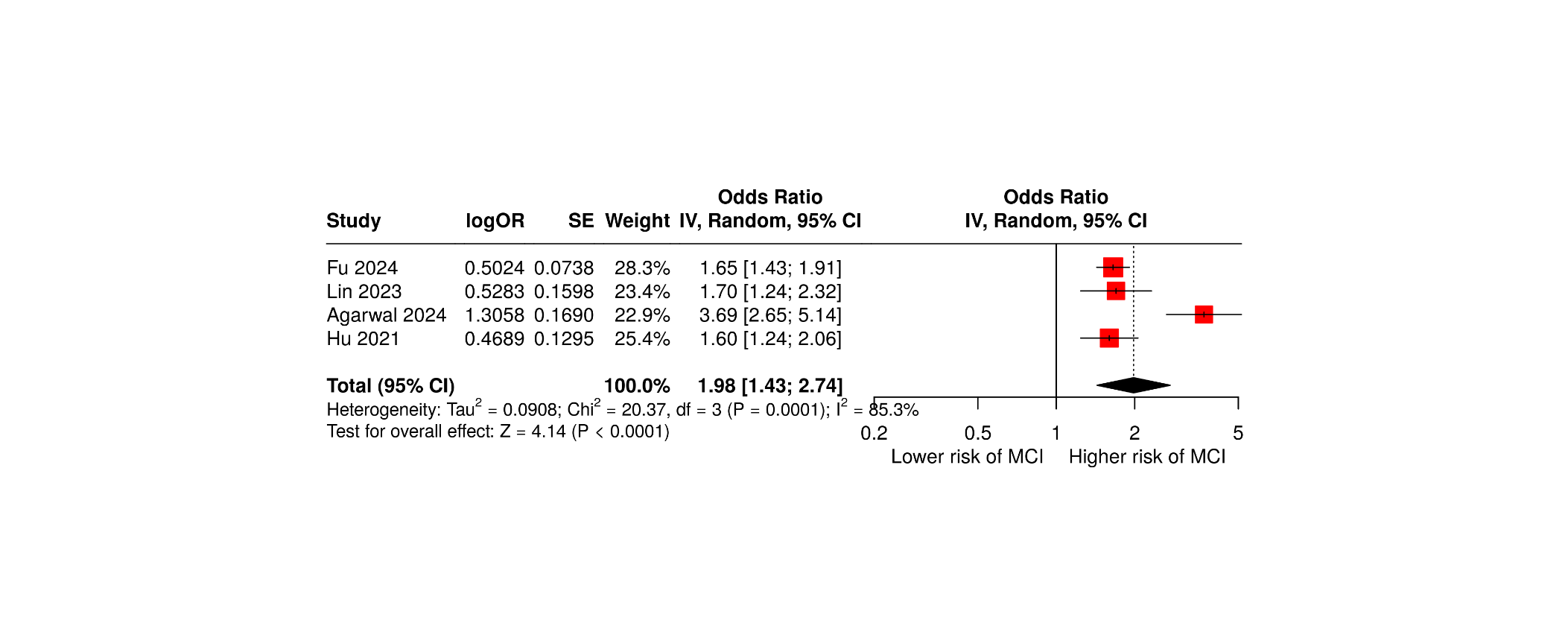


**Supplementary Figure 9.** Forest plot of the association between periodontitis + tooth loss and mild cognitive impairment.

MCI: mild cognitive impairment


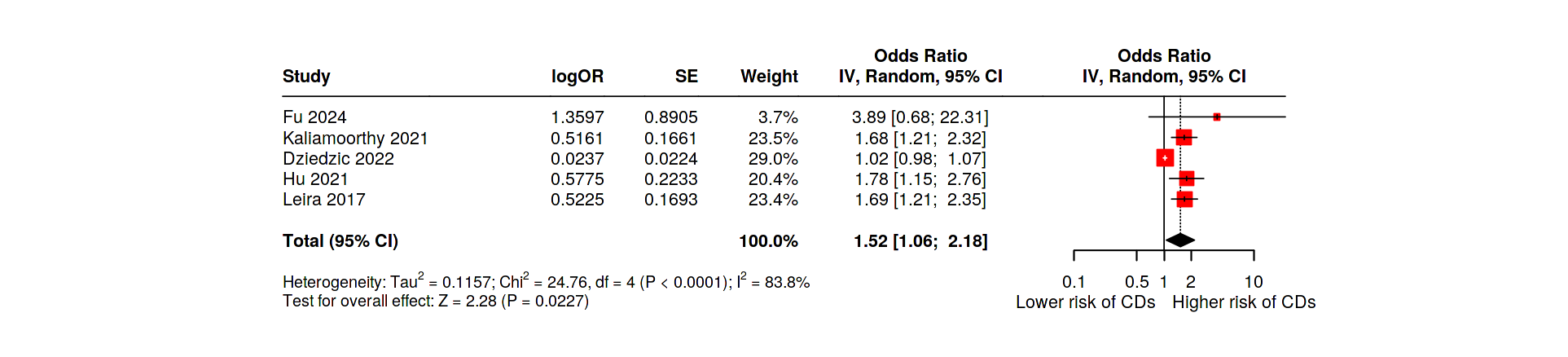


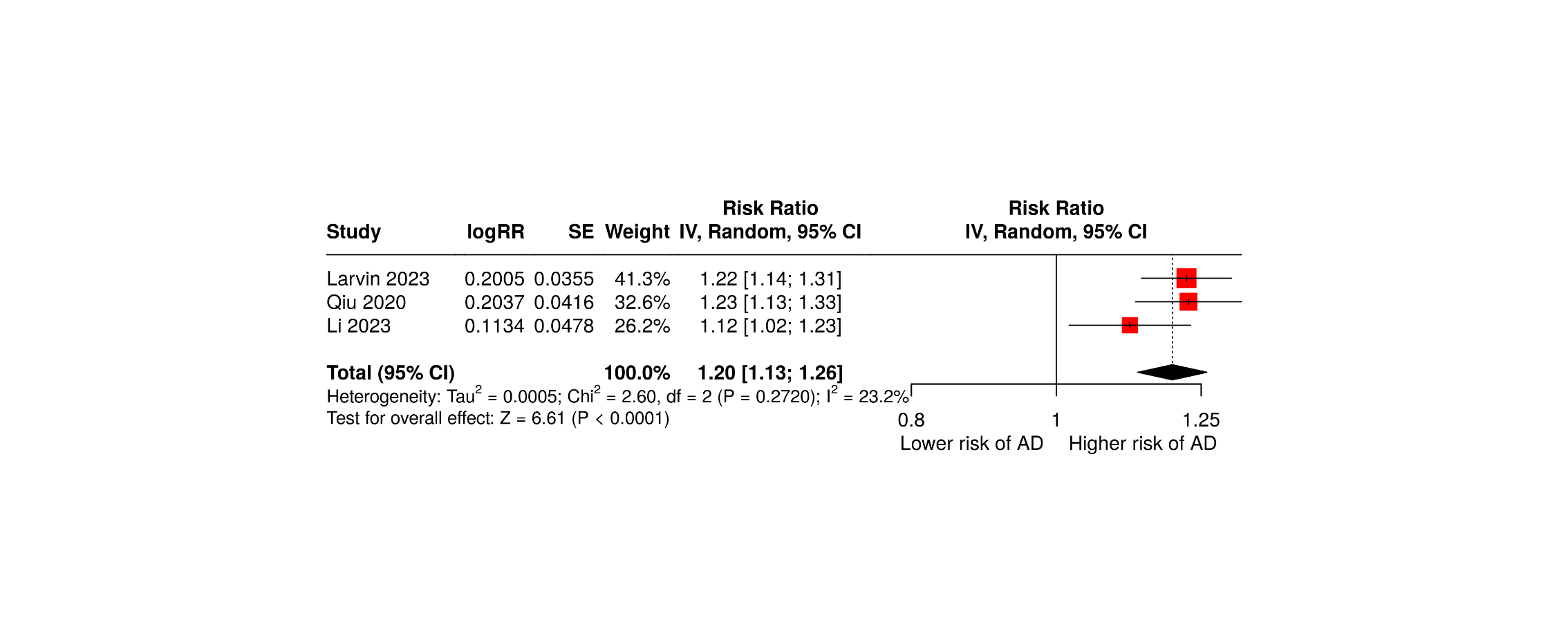


**Supplementary Figure 10.** Forest plot of the association between periodontitis + tooth loss and Alzheimer’s disease in OR and RR.

AD: Alzheimer’s disease

**References**

1. Agarwal B, Bizzoca ME, Musella G, De Vito D, Lo Muzio L, Ballini A, et al. Tooth Loss in Periodontitis Patients—A Risk Factor for Mild Cognitive Impairment: A Systematic Review and Meta—Analysis. J Pers Med. 2024;14(9):953.

2. Asher S, Stephen R, Mäntylä P, Suominen AL, Solomon A. Periodontal health, cognitive decline, and dementia: A systematic review and meta‐analysis of longitudinal studies. J Am Geriatr Soc. 2022;70(9):2695–709.

3. Chen J, Ren CJ, Wu L, Xia LY, Shao J, Leng WD, et al. Tooth loss is associated with increased risk of dementia and with a dose-response relationship. Front Aging Neurosci. 2018;10:415.

4. Dibello V, Custodero C, Cavalcanti R, Lafornara D, Dibello A, Lozupone M, et al. Impact of periodontal disease on cognitive disorders, dementia, and depression: a systematic review and meta-analysis. Geroscience. 2024;46(5):5133–69.

5. Dziedzic A. Is periodontitis associated with age-related cognitive impairment? The systematic review, confounders assessment and meta-analysis of clinical studies. Int J Mol Sci. 2022;23(23):15320.

6. Fang W li, Jiang M jun, Gu B bei, Wei Y mei, Fan S nuo, Liao W, et al. Tooth loss as a risk factor for dementia: systematic review and meta-analysis of 21 observational studies. BMC Psychiatry. 2018;18:1–11.

7. Fu YD, Li CL, Hu CL, Pei MD, Cai WY, Li YQ, et al. Meta Analysis of the Correlation between Periodontal Health and Cognitive Impairment in the Older Population. J Prev Alzheimers Dis. 2024;11(5):1307–15.

8. Guo H, Chang S, Pi X, Hua F, Jiang H, Liu C, et al. The effect of periodontitis on dementia and cognitive impairment: a meta-analysis. Int J Environ Res Public Health. 2021;18(13):6823.

9. Hu X, Zhang J, Qiu Y, Liu Z. Periodontal disease and the risk of Alzheimer’s disease and mild cognitive impairment: a systematic review and meta‐analysis. Psychogeriatrics. 2021;21(5):813–25.

10. Kaliamoorthy S, Nagarajan M, Sethuraman V, Jayavel K, Lakshmanan V, Palla S. Association of Alzheimer’s disease and periodontitis-a systematic review and meta-analysis of evidence from observational studies. Med Pharm Rep. 2022;95(2):144.

11. Kim DH, Han GS. Periodontitis as a risk factor for dementia: a systematic review and meta-analysis. J Evid-Based Dent Pract. 2025;102094.

12. Larvin H, Gao C, Kang J, Aggarwal VR, Pavitt S, Wu J. The impact of study factors in the association of periodontal disease and cognitive disorders: systematic review and meta-analysis. Age Ageing. 2023;52(2):afad015.

13. Leira Y, Dominguez C, Seoane J, Seoane-Romero J, Pías-Peleteiro JM, Takkouche B, et al. Is periodontal disease associated with Alzheimer’s disease? A systematic review with meta-analysis. Neuroepidemiology. 2017;48(1–2):21–31.

14. Li L, Zhang Q, Yang D, Yang S, Zhao Y, Jiang M, et al. Tooth loss and the risk of cognitive decline and dementia: A meta-analysis of cohort studies. Front Neurol. 2023;14:1103052.

15. Lin CS, Chen TC, Verhoeff MC, Lobbezoo F, Trulsson M, Fuh JL. An umbrella review on the association between factors of oral health and cognitive dysfunction. Ageing Res Rev. 2024;93:102128.

16. Nadim R, Tang J, Dilmohamed A, Yuan S, Wu C, Bakre AT, et al. Influence of periodontal disease on risk of dementia: a systematic literature review and a meta-analysis. Eur J Epidemiol. 2020;35:821–33.

17. Qi X, Zhu Z, Plassman BL, Wu B. Dose-response meta-analysis on tooth loss with the risk of cognitive impairment and dementia. J Am Med Dir Assoc. 2021;22(10):2039–45.

18. Qiu C, Zhou W, Shi WT, Song ZC. Association between periodontitis and Alzheimer disease: a meta analysis. Shanghai Kou Qiang Yi Xue Shanghai J Stomatol. 2020;29(6):661–8.

19. Raymundo MLB, da Silva RO, de Araújo ECF, de Lucena EHG, Cavalcanti YW. Is the history of periodontal disease a risk factor for cognitive decline? Systematic Review and Meta-analysis. Res Soc Dev. 2022;11(5):e15811527931–e15811527931.

20. Shen T, Lv J, Wang L, Wang W, Zhang D. Association between tooth loss and dementia among older people: a meta‐analysis. Int J Geriatr Psychiatry. 2016;31(8):953–5.
